# Comprehensive Exonic Sequencing of Hemiplegic Migraine-Related Genes in a Cohort of Suspected Probands Identifies Known and Potential Pathogenic Variants

**DOI:** 10.3390/cells9112368

**Published:** 2020-10-28

**Authors:** Heidi G. Sutherland, Neven Maksemous, Cassie L. Albury, Omar Ibrahim, Robert A. Smith, Rod A. Lea, Larisa M. Haupt, Bronwyn Jenkins, Benjamin Tsang, Lyn R. Griffiths

**Affiliations:** 1 Centre for Genomics and Personalised Health, Genomics Research Centre, School of Biomedical Sciences, Institute of Health and Biomedical Innovation, Queensland University of Technology (QUT), Brisbane, QLD 4059, Australia; heidi.sutherland@qut.edu.au (H.G.S.); n.maksemous@qut.edu.au (N.M.); calbury15@hotmail.com (C.L.A.); omar.abdelrahman@hdr.qut.edu.au (O.I.); r157.smith@qut.edu.au (R.A.S.); rodney.lea@qut.edu.au (R.A.L.); larisa.haupt@qut.edu.au (L.M.H.); 2 The Epping Clinic, Sydney, NSW 2121, Australia; bronwyn.jenkins@icloud.com; 3 Department of Neurology, Sunshine Coast University Hospital, Birtinya, QLD 4575, Australia; benjamin.tsang@health.qld.gov.au

**Keywords:** familial hemiplegic migraine, migraine, paroxysmal movement disorders, whole exome sequencing, ion channel genes, mutations, variants

## Abstract

Hemiplegic migraine (HM) is a rare migraine disorder with aura subtype including temporary weakness and visual, sensory, and/or speech symptoms. To date, three main genes—*CACNA1A*, *ATP1A2*, and *SCN1A*—have been found to cause HM. These encode ion channels or transporters, important for regulating neuronal ion balance and synaptic transmission, leading to HM being described as a channelopathy. However, <20% of HM cases referred for genetic testing have mutations in these genes and other genes with roles in ion and solute transport, and neurotransmission has also been implicated in some HM cases. In this study, we performed whole exome sequencing for 187 suspected HM probands referred for genetic testing, but found to be negative for *CACNA1A*, *ATP1A2*, and *SCN1A* mutations, and applied targeted analysis of whole exome sequencing data for rare missense or potential protein-altering variants in the *PRRT2*, *PNKD*, *SLC1A3*, *SLC2A1*, *SLC4A4*, *ATP1A3*, and *ATP1A4* genes. We identified known mutations and some potentially pathogenic variants in each of these genes in specific cases, suggesting that their screening improves molecular diagnosis for the disorder. However, the majority of HM patients were found not to have candidate mutations in any of the previously reported HM genes, suggesting that additional genetic factors contributing to the disorder are yet to be identified.

## 1. Introduction

Hemiplegic migraine (HM) is a rare migraine disorder with aura subtype including temporary weakness, usually affecting one side of the body, and visual, sensory, and/or speech symptoms. There are sporadic and familial forms of HM (SHM and FHM, respectively), with the latter usually showing autosomal dominant inheritance [1]. Studies of HM families have led to the identification of three main causal genes, *CACNA1A*, *ATP1A2*, and *SCN1A,* which encode ion channel or ion transport proteins; functional analysis of HM mutations have demonstrated that they can result in defective regulation of glutamatergic neurotransmission and the excitatory/inhibitory balance in the brain [2]. However, many familial and sporadic cases do not appear to have causal mutations in these main FHM genes [3,4].

In addition to the three FHM genes, other genes with roles in synaptic function and neurotransmission have also been implicated in HM and HM-like disorders, although the evidence for some is more limited. Mutations in *PRRT2* have been reported to cause HM and it was proposed as the fourth HM gene, although the relationship is complicated due to clinical heterogeneity and pleiotropy of phenotypes [5,6,7,8]. *PRRT2* mutations can cause paroxysmal movement disorders, including paroxysmal kinesigenic dyskinesia (PKD) [9,10], some cases of paroxysmal non-kinesigenic dyskinesia (PNKD), and paroxysmal exercise-induced dyskinesia (PED) [11], as well as the childhood epilepsy/seizure disorders benign familial infantile epilepsy (BFIE) and infantile convulsions and choreoathetosis (ICCA) syndrome [12,13]. PRRT2 is a presynaptic transmembrane protein that interacts with members of the SNAP receptor (SNARE) complex and is involved in synaptic vesicle fusion and regulation of voltage-gated calcium channels in glutamatergic neurons [14,15]. The *PRRT2* c.649dupC (p.Arg217Profs*8) or c.649delC (p.Arg217Glufs*12) loss of function truncating mutations are the most common reported for PRRT2-related disorders [7], occurring at a mutational hotspot homopolymer of four guanine bases followed by nine consecutive cytosines that can expand or contract to result in frameshifts. These, and many of the other reported *PRRT2* mutations, lead to an unstable messenger RNA or truncated protein that undergoes rapid degradation, usually via nonsense mediated decay [16], resulting in haploinsufficiency and consistent with a loss-of-function mechanism. Some mutations may also affect protein localisation or interactions and are likely to impair association with the SNAP25/SNARE complex, leading to greater presynaptic vesicle release [11].

**Figure 1:**
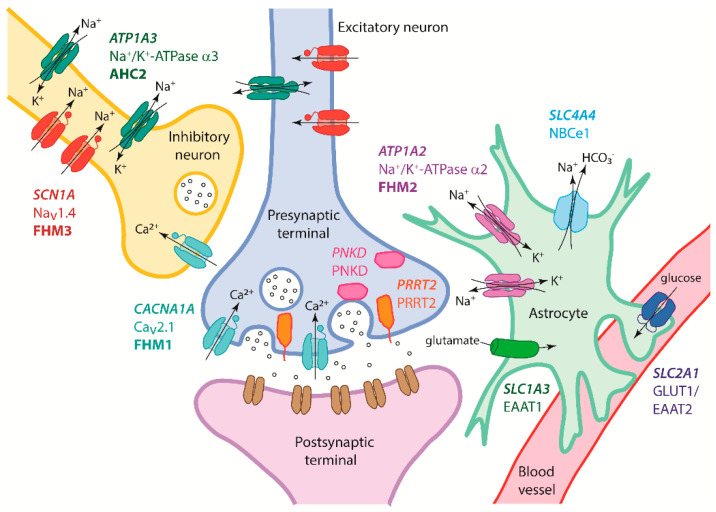
Genes and their encoded proteins involved in hemiplegic migraine (HM) shown at a tripartite glutamatergic synapse in the central nervous system (adapted from Russell and Ducros [1]). A presynaptic excitatory neuron along with an inhibitory gamma aminobutyric acid (GABA)ergic interneuron is shown with a postsynaptic neuron, their surrounding astrocyte, and an associated blood vessel. The three major HM genes—*CACNA1A*, *ATP1A2*, and *SCN1A*—cause FHM1, FHM2, and FHM3, respectively. Cav2.1 channels encoded by *CACNA1A* are located in the presynaptic terminal of excitatory and inhibitory neurons and, in response to an action potential, allow entry of Ca^2+^, triggering glutamate release into the synaptic cleft. *ATP1A2* encodes the Na^+^-K^+^ ATPase α2 pump subunit, expressed on the surface of astrocytic glial cells, and removes K^+^ from the synaptic cleft to limit neuronal excitability and maintain a Na^+^ gradient across the cell membrane. Nav1.1 channels, encoded by *SCN1A*, are mainly expressed on GABAergic neurons and inhibitory interneurons, mediating voltage-dependent Na^+^ influx and regulating their excitability. The PRRT2 protein encoded by *PRRT2* is localised at glutamatergic synapses where it interacts with proteins (e.g., SNAP25 and Ca^2+^ sensors Syt1/2) to mediate the activation of fast and synchronous neurotransmitter release. Similarly, paroxysmal non-kinesigenic dyskinesia (PNKD) protein encoded by *PNKD* interacts with synaptic active zone proteins (e.g., RIM1 and RIM2) to modulate neurotransmitter release. *SLC4A4* encodes the electrogenic Na^+^-HCO^3^ cotransporter NBCe1, with some isoforms expressed in the brain and on glial cells, which play a role in regulation of synaptic pH and neurotransmission. *ATP1A3* encodes the Na^+^-K^+^ ATPase α3 pump subunit, which functions to maintain the electrochemical gradient across neuronal membranes to regulate their excitability and has a likely role at inhibitory synapses. Mutations in *ATP1A3* can cause alternating hemiplegia of childhood (AHC). EAAT1 encoded by *SLC1A3* is a Na^+^/K^+^-dependent glutamate transporter that recaptures glutamate from the synaptic cleft into glial cells including astrocytes to terminate its postsynaptic action. *SLC2A1* encodes the GLUT1 glucose transporter and is present at the blood–brain barrier to facilitate glucose transport into the brain. Na^+^, sodium ion; K^+^, potassium ion; Ca^2+^, calcium ion.

As well as *PRRT2*, mutations in other synaptic, ion channel, and transport genes have been reported to cause HM in some cases and include *PNKD* [11], the main gene implicated in PNKD [17,18]; *SLC2A1*, encoding the glucose transporter protein type 1 (GLUT1 or EAAT2) [19], in which mutations commonly cause GLUT1 deficiency syndrome [20]; *SLC1A3*, encoding the glial glutamate transporter EAAT1 [21], in which mutations can cause episodic ataxia, type 6 [22]; and *SLC4A4*, encoding the sodium bicarbonate cotransporter NBCe1 [23], in which causal mutations have also been found in renal tubular acidosis (RTA) syndromes [24]. Recently, *ATP1A4*, which encodes Na^+^/K^+^ ATPase alpha 4, has also been suggested as a new FHM gene with the detection of a mutation segregating the disease phenotype in a single Italian family [25]. There are also genes in which mutations can cause neurological disorders that may have overlapping symptoms with HM, e.g., mutations in *ATP1A3*, which encodes Na^+^/K^+^ ATPase alpha 3, can cause alternating hemiplegia of childhood (AHC) [26], and mutations in *NOTCH3* cause cerebral autosomal dominant arteriopathy with subcortical infarcts and leukoencephalopathy (CADASIL), with patients often presenting with migraine or hemiplegic migraine before manifestation of other symptoms [27,28]. The proteins encoded by the three main FHM genes, as well as the majority of other genes previously implicated in HM, have roles at tripartite synapses and the supporting astrocytes and are important in synaptic transmission at glutamatergic neurons and regulating the excitatory-inhibitory balance in the brain (Figure 1).

We have developed a customised diagnostic next generation sequencing (NGS) panel that includes full exonic coverage of the FHM genes *CACNA1A*, *ATP1A2*, and *SCN1A*. Using this panel, we identified 29 different mutations (12 known and 17 novel, predominantly in *ATP1A2* and *CACNA1A*) in 35 cases of 172 suspected hemiplegic migraineurs referred to our laboratory for diagnostic testing [4]. However, including all other patients and mutations we have previously identified via Sanger sequencing, we found that 18.7% of patients referred for HM genetic testing were positive for mutations in the three main FHM genes, suggesting that variants in other genes may cause HM symptoms in many cases.

In this study, we investigated whether a cohort of probands from Australia and New Zealand referred for FHM gene testing—who were found to be negative for mutations in *CACNA1A*, *ATP1A2*, and *SCN1A*—had likely pathogenic variants in any of the other genes previously implicated in HM. Sanger sequencing was used to assess presence of the most common *PRRT2* mutations (the loss of function frameshifts c.649dupC or c.649delC). Whole exome sequencing (WES) was performed on the cohort and targeted analysis of data was conducted to identify variants in the remaining *PRRT2* coding exons and other HM-related genes (*PNKD*, *SLC1A3, SLC2A1*, *SLC4A4, ATP1A3*, and *ATP1A4*). We identified some known mutations, as well as potentially pathogenic variants, in these genes in specific cases, improving the diagnostic rate. However, the majority of patients did not have candidate mutations in any of the known HM genes, thereby suggesting that additional genetic factors that contribute to the disorder are yet to be identified.

## 2. Materials and Methods

### 2.1. Patients

Blood samples from individuals from Australia and New Zealand suspected of having hemiplegic migraine after diagnosis by a neurologist were sent to our laboratory for diagnostic testing of the FHM genes. All cases consented to genetic testing with their doctors, as required under current regulations. This study was approved by the Queensland University of Technology (QUT) Ethics Committee (approval number: 1800000611). A family history was reported for 25% of the cases, 5% were reported as SHM, while family information was not provided for the remainder. For these patients, comprehensive sequencing of *CACNA1A*, *ATP1A2*, and *SCN1A* exons, flanking intronic sequences, and 5′ and 3′ untranslated regions, was conducted with extracted DNA samples using a targeted 5-gene panel (which also includes *NOTCH3* and *KCNK18*) on the Ion Torrent next-generation sequencing (NGS) platform [29]. In ~80% of the samples, no likely pathogenic variants were identified in any of the genes screened [4]. A total of 187 such cases, negative for pathogenic variants in *CACNA1A*, *ATP1A2*, and *SCN1A*, were further examined in this study.

Characteristics including sex, age, and any noted family history, as well as available clinical details, of cases with rare variants identified in the present study are shown in Supplementary Table S1. Due to the nature of this diagnostic cohort, for many cases only limited clinical information was available beyond a suspected diagnosis of hemiplegic migraine.

### 2.2. Molecular Analysis of the PRRT2 Mutation Hotspot

Sanger sequencing was used to investigate whether mutations in *PRRT2*, in particular c.649dupC (p.Arg217Profs*8) and variants around this hotspot, were present in the 187 suspected HM cases. Sanger sequencing was the method of choice for this gene as it can be difficult to reliably call *PRRT2* hotspot mutations on NGS platforms due to the associated homopolymer stretch. A 334 bp PCR product encompassing the majority of exon 2 was generated using the primers F: 5′AAGAGAATGGGGCAGTGGTG and R: 5′ TAAGCGAAGGCCACGATGTT. Amplified products were treated with shrimp alkaline phosphatase (Thermo Fisher Scientific, Scoresby, Victoria, Australia) and sequencing reactions performed using forward and reverse primers and Big Dye v3.1 (Thermo Fisher Scientific), followed by separation on an ABI 3500 genetic analyser (Thermo Fisher Scientific), according to the manufacturer’s instructions.

### 2.3. Whole Exome Sequencing of the HM Cohort

WES was performed on the 187 HM cases using the Ion Torrent platform as previously described [30]. Briefly, WES libraries were prepared using the Ion AmpliSeq Exome RDY library preparation kit (catalogue number: A38264, Revision C.0, Thermo Fisher Scientific, Scoresby, Australia) according to the manufacturer’s protocol and sequenced on the Ion Proton sequencer (Thermo Fisher Scientific). Reads were aligned to the human reference genome (hg19) and single-nucleotide variants and indels called using Ion Torrent Suite software. BAM files generated by the Torrent Suite were uploaded and visualised using the Broad Institute Integrative Genomics Viewer (IGV) 2.3, and locally hosted Ion Reporter software 4.10 (Life Technologies) was used to perform automated variant annotation and filtering.

### 2.4. Molecular Analysis of Genes Previously Implicated in HM in WES Data of Cohort

To assess whether potentially pathogenic variants were present in the remainder of the *PRRT2* exons, as well as protein-coding regions for other genes previously associated with HM or related conditions—*PNKD*, *SLC1A3*, *SLC4A4*, *SLC2A1*, *ATP1A3*, and *ATP1A4*—we performed targeted analysis of WES data generated for the cohort. Annotated variants were filtered for those that were potentially functional, including non-synonymous, frameshifts, stop-gain or losses, or in canonical splice sites, and with a minor allele frequency of <1% in general populations (gnomAD), noting frequencies of variants in both the total gnomAD population as well as the European non-Finnish population (most relevant for our cohort). These filtered variants were then further assessed for pathogenicity using in silico tools including Sorting Intolerant From Tolerant (SIFT, https://sift.bii.a-star.edu.sg/), Polymorphism Phenotyping v2 (Polyphen2, http://genetics.bwh.harvard.edu/pph2/), and MutationTaster (http://www.mutationtaster.org/), as well as nucleotide conservation scores derived from PhyloP and PhastCons, and information from clinical databases (ClinVar, Leiden Open Variation Database v3.0 (LOVD3)) and the literature.

## 3. Results

### 3.1. Screening of HM Cases for Variants around the PRRT2 Mutation Hotspot by Sanger Sequencing

The study cohort consisted of 187 suspected HM cases from Australia and New Zealand referred to our laboratory for molecular genetic testing, found negative for pathogenic exonic or splice site mutations in the main HM genes *CACNA1A*, *ATP1A2*, and *SCN1A*. Furthermore, as the cases were screened by our custom five-gene panel, they had also been assessed for mutations in the CADASIL gene, *NOTCH3*, and *KCNK18*, in which mutations can cause familial migraine with aura [31]; although some non-cysteine altering variants in *NOTCH3* were detected (manuscript in preparation), no known mutations in these genes were identified.

**Figure 2:**
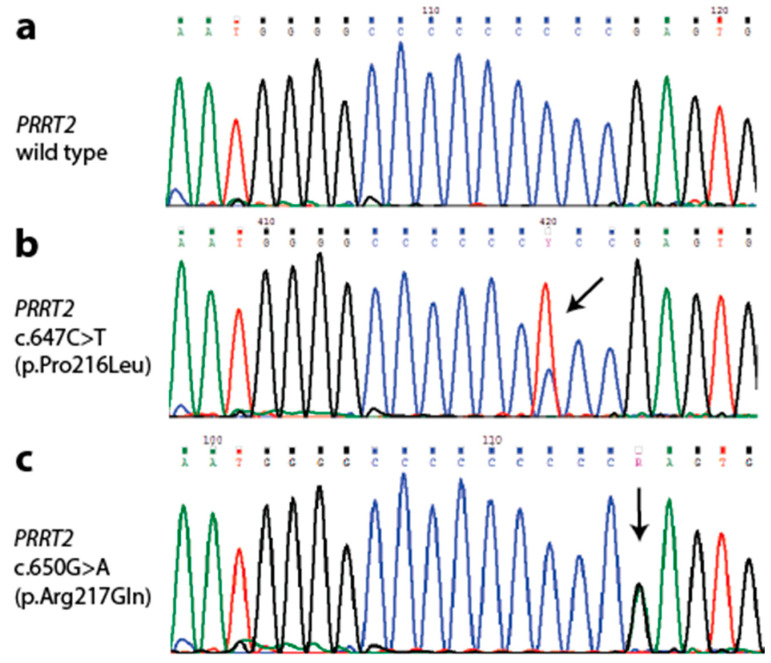
Sanger sequencing traces of *PRRT2* mutation hotspot region. (**a**) Wild type PRRT2 around the mutation hotspot detected in 182 HM cases. (**b**) *PRRT2* c.647C>T (Pro216Leu) missense variant detected in HM cases 185, 201, 211, and 227. (**c**) *PRRT2* c.650G>A (p.Arg217Gln) missense variant in HM case 307. Arrow indicates variant.

To firstly determine whether any individuals in the cohort carried c.649dupC (p.Arg217Profs*8), c.649delC (p.Arg217Glufs*12), or c.650delG (p.Arg217Glnfs*12) mutations, we screened DNA samples from each case for mutations around the *PRRT2* cytosine homopolymer mutation hotspot in exon 2 by Sanger sequencing (Figure 2). While all the previously reported HM cases with a *PRRT2* mutation had one of these [11], none were detected in our cohort. However, within this region, four HM cases (ID# 185, 201, 211, and 227) had a c.647C>T (Pro216Leu) missense variant (rs76335820) and one case (433) carried a c.650G>A (Arg217Gln) variant (Table 1, Figure 2). The Pro216Leu variant was predicted to be damaging/disease causing using the in silico prediction tools SIFT, Polyphen2, and MutationTaster; however, it is moderately common in population databases (minor allele frequency [MAF] ~0.7–1% in gnomAD). The Arg217Gln variant (rs75497546) is very rare and predicted to be damaging by some in silico tools (SIFT, Polyphen2) (Table 1). Notably, case 433 also carries an *ATP1A2* (p.Gly114Ser) variant, but was included in this study as the *ATP1A2* variant had been assessed as being of unknown significance by American College of Medical Genetics (AMCG) guidelines [32].

### 3.2. Examination of Whole Exome Sequencing (WES) Data for Pathogenic Variants in HM-Related Genes

WES using the Ion Torrent NGS platform was performed for the cohort of 187 cases. The available WES data were assessed by targeted analysis for variants in the remainder of the *PRRT2* exons (as the Sanger sequencing amplicon did not encompass the complete coding region of the gene), as well as the other genes previously implicated in HM and related conditions (*PNKD*, *SLC1A3*, *SLC2A1*, *SLC4A4*, *ATP1A3*, and *ATP1A4*). Data were filtered for rare potentially protein-altering variants (gnomAD allele frequency <1%) consisting of those that result in non-synonymous amino acid changes, truncations, or are predicted to affect splicing. Sanger sequencing was used to validate identified variants, particularly any with low coverage or unclear in the Integrative Genomics Viewer (IGV, Broad Institute). Variants of interest were identified in each of the genes, and are shown in Table 1, along with annotations of dbSNP number, allele frequency, and in silico predictions of pathogenicity and conservation. In addition to *PRRT2* variants around the mutation hotspot, we identified two unrelated individuals (ID# 224 and 225) to have a c.67G>A (p.Glu23Lys) missense variant predicted to be benign that has also been annotated as a variant of unknown significance (VUS) in ClinVar, and one case (137) had a very rare c.988G>A (p.Ala330Thr) variant predicted to be disease-causing by MutationTaster. We also identified a rare C>T change at chr16:29825888 in HM case 261, which results in a c.1114C>T (p.Leu372Phe) missense variant (rs565298585, allele frequency = 0.00005 in gnomAD), predicted to be damaging by SIFT, but benign by Polyphen2 and neutral by MutationTaster. It should be noted that this variant is only included in the *PRRT2* coding sequence in an isoform in which intron 3 is retained to produce an extended protein compared with the canonical isoform usually associated with PRRT2-related disorders. All *PRRT2* rare variants were confirmed by Sanger sequencing.

Interrogation of WES data for *PNKD* variants found a single rare 6 base pair (bp) deletion in case 32 (c.1140_1145delTATGCA; p.Met381_His382del), which results in an in-frame deletion of two amino acids from the protein. For the *SLC1A3* gene, one patient (107) had a rare c.1154G>A (p.Arg385His) variant at a highly conserved amino acid that was predicted to be damaging by multiple in silico tools. In addition, four cases (135, 155, 159, and 196) were identified with a c.657G>C p.Glu219Asp *SLC1A3* variant with an allele frequency of 0.7% in the European Non-Finnish gnomAD population, but that was more common in some gnomAD populations.

Targeted analysis of *SLC2A1* identified one known variant and two potentially disease-causing variants: the c.929C>T (p.Thr310Ile) mutation found in case 189 has previously been reported to cause GLUT1 deficiency [33]; a *SLC2A1* c.203C>T (p.Ser68Leu) variant was found in patient 109, which is absent from gnomAD, but reported in ClinVar as a VUS for GLUT1 deficiency; and an intronic variant (c.972+7del; p.Leu232PhefsTer?) predicted by MutationTaster to affect splicing was identified in case 179. Notably, this case also has a *SLC4A4* (p.Lys602Arg) variant (see below).

Rare missense heterozygous variants in the *SLC4A4* gene were detected in numerous cases in the cohort, with some occurring in two unrelated cases (Table 1). There were five variants found in one case each: c.313C>G (p.Pro105Ala), c.898A>G (p.Ile300Val), c.2311C>T (p.Pro771Ser), c.2485C>A (p.Leu829Ile), and the novel variant c.1229G>A (p.Gly410Glu). A c.1805A>G (p.Lys602Arg) missense variant was present in two unrelated individuals (120 and 179). This variant (rs72650362) is moderately rare with an allele frequency of 0.002 in gnomAD and is predicted to be damaging or disease-causing by the three in silico tools used and is highly conserved (PhyloP 5.00, PhastCons 1). Case 179 also had the *SLC2A1* variant mentioned previously. Another two unrelated cases (202 and 232) both bore a *SLC4A4* c.2051A>T (p.Asn684Ile) variant.

*ATP1A3* encodes a neuronally expressed Na^+^/K^+^ ATPase in the same family as *ATP1A2*. Targeted analysis of this gene revealed two known mutations for alternating hemiplegia of childhood: c.2401G>A (p.Asp801Asn) in case 165 and c.2443G>A (p.Glu815Lys) in case 87 [26]. Furthermore, as *ATP1A4* was recently reported as a tentative hemiplegic migraine gene [25], we also performed targeted analysis of WES data for rare functional variants in this gene. Numerous missense variants in *ATP1A4* were identified in the cohort (Table 1). A total of 12 of the 187 cases had at least one such variant, including case 186 with two variants, and case 144 with three.

## 4. Discussion

*CACNA1A*, *ATP1A2*, and *SCN1A* are the major genes in which mutations have been found to cause HM. However, ∼80% of the patients referred for genetic testing remain without a molecular diagnosis, despite full exonic sequencing for these genes [4]. In this study, we report a large screen by exonic sequencing of patients referred for FHM gene testing for mutations or rare variants in the remaining genes previously implicated in HM and related disorders (*PRRT2*, *PNKD*, *SLC1A3, SLC2A1*, *SLC4A4, ATP1A3*, and *ATP1A4*). We identified some known mutations, as well as other potentially disease-causing variants.

*PRRT2* has been proposed as a fourth HM gene and *PRRT2* mutations have been found in individuals who predominantly show symptoms of HM [5,6,7,11], rather than the other paroxysmal disorders they are commonly associated with [9,10]. Sanger sequencing (Figure 2) showed that none of the cases with suspected HM in this study had the c.649dupC (p.Arg217Profs*8) or c.649delC (p.Arg217Glufs*12) loss of function truncating mutations most commonly found in *PRRT2*-related disorders and HM cases [5,6,7,11]. Our results add strength to findings by Pelzer et al. that *PRRT2* mutations are not common in HM patients without *CACNA1A*, *ATP1A2*, and *SCN1A* mutations [34], and that assigning causality to *PRRT2* variants may be complicated [35]. Five different missense *PRRT2* variants were identified in our cohort of index HM cases. Two unrelated cases (224 and 225), both young children (Table S1), had the same rare *PRRT2* c.67G>A (p.Glu23Lys) variant, which has been annotated as either likely benign or a VUS in ClinVar. Case 433 had a p.Arg217Gln missense variant in *PRRT2*, due to a c.650G>A base substitution rather than an insertion or deletion. This variant (MAF = 0.000008 in gnomAD) has not been previously reported for *PRRT2*-related diseases and its pathogenicity remains unclear. The case was also identified to have an *ATP1A2* p.Gly114Ser variant [4], but was included in this study as it was reported as a variant of unknown significance (VUS) on the basis of pathogenicity predictions and frequency, according to ACMG Guidelines [32]. It is possible the combination of both the *ATP1A2* and *PRRT2* variants contribute to expression of HM symptoms, with the latter having a genetic modifier role as suggested by Pelzer et al. [35]. A recent finding that PRRT2 is a negative modulator of some Na^+^ ion channels in addition to synaptic functions [15] further suggests that variants in *PRRT2* could interact with those in other genes in overlapping pathways.

Four of the 187 cases (2.1%) had the same *PRRT2* c.647C>T (p.Pro216Leu) variant. However, while predicted to by damaging by some in silico tools, it is unlikely to be causal as it is relatively common in large general populations (0.7% in gnomAD, 1.0% in non-Finnish Europeans). Furthermore, this variant was detected at a similar frequency (5.2%) in an Australian control population investigating *PRRT2* in BFIE and ICCA [12], and did not segregate with disease phenotype in a number of PKD pedigrees [36]. A rare p.Leu372Phe variant in a region of the protein with low conservation, and not present in the main splice forms, was present in case 261. Although unlikely to be solely pathogenic, variants annotated as benign may nevertheless modulate the function of variants that may be present in other genes yet to be identified.

Apart from *PRRT2*, we also detected both known mutations and variants of interest in other genes previously implicated in HM. PNKD is another protein with a role in regulating neurotransmitter release at the synapse, in which mutations are mainly associated with paroxysmal movement disorders, but can cause hemiplegic migraine in some patients [11]. Only one rare variant in *PNKD* was detected in the cohort—case 32 had a rare in-frame deletion of two amino acids, c.1140_1145delTATGCA (p.Met381_His382del), although this is suggested to be likely benign in ClinVar.

For the glial glutamate transporter EAAT1 encoded by *SLC1A3*, we identified a c.1154G>A (p.Arg385His) variant at a highly conserved amino acid in the transport domain of the protein, predicted to be damaging by multiple in silico tools. While only a few hemiplegic migraine cases have been reported with *SLC1A3* variants, one of these had a p.Thr387Pro change in this same region of the protein, which resulted in a loss of function effect on the transporter, impairing both K^+^ binding as well as its trafficking to the membrane [21]. Four cases had the same *SLC1A3* c.657G>C (p.Glu219Asp) variant, which has been shown to have a gain of function effect on the transporter [37]. However, as this variant is relatively common in gnomAD (Table 1), particularly in some populations (e.g., Latino), it is unlikely to be solely causal.

Mutations in *SLC2A1*, which encodes the glucose transporter GLUT1, most commonly cause de vivo or GLUT1 deficiency syndrome (GLUT1 DS), characterized by infantile seizures, acquired microcephaly, developmental delay, and motor incoordination consisting of ataxia and dystonia (OMIM #606777). Patients usually have a low cerebrospinal fluid to blood glucose ratio (<0.5), decreased erythrocyte glucose uptake, and impaired facilitative glucose transport across the blood-brain barrier [38]. The rare *SLC2A1* p.Thr310Ile variant identified in case 189 in this study is a known mutation, previously reported in a number of GLUT1 deficiency pedigrees, and has been shown to functionally impair glucose transport [33]. Thr310 forms part of a network of hydrogen bonds between the N and C domains that occludes the ligand-binding site from the extracellular environment in the inward-facing conformation of GLUT1 [39]. Follow-up on this patient confirmed that while HM was the dominant presenting clinical feature, they had GLUT1 deficiency syndrome and the p.Thr310Ile mutation was de novo [40]. Potentially pathogenic *SLC2A1* variants were identified in two other cases. Case 109 has a *SLC2A1* c.203C>T (p.Ser68Leu) variant, which is absent from general population databases, but annotated as a VUS in ClinVar for GLUT1 DS; the mutated amino acid is directly adjacent to residues critical for glucose recognition and binding [41]. Case 179 has an intronic variant (c.972 + 7del) predicted by MutationTaster to affect splicing, which is very rare in the general population, but has been reported multiple times in mutation databases with conflicting interpretations of pathogenicity.

Homozygous mutations in *SLC4A4* encoding the sodium bicarbonate cotransporter NBCe1 are usually associated with proximal renal tubular acidosis, with ocular abnormalities (RTA, OMIM #604278), which can also feature mental retardation and short stature. Some *SLC4A4* mutations, which result in defective or impaired trafficking of the transporter to the plasma membrane in astrocytes, have been implicated in HM [23]. Multiple variants of interest in *SLC4A4* were found in our HM testing cohort. Unrelated cases 120 and 179 were both found to have same heterozygous c.1805A>G (p.Lys602Arg) missense variant. Previous functional testing of Lys602, which resides in the largest extracellular loop of NBCe1, found that its mutation has a moderate effect on transporter activity (50% of wild type) and suggested that it may assist neighbouring lysines in HCO3^−^ recognition [42]. As the variant is moderately common in the general population (gnomAD frequency of 0.002), it would likely have a modifying role if any. Two other unrelated cases (202 and 232) both had a *SLC4A4* c.2051A>T (p.Asn684Ile) variant, also with a gnomAD frequency of ~0.002. Case 237 had a novel variant, c.1229G>A (p.Gly410Glu), while four other cases (136, 142, 128, and 150) each had different rare *SLC4A4* variants; the p.Pro771Ser and p.Leu829Ile changes in the latter two cases have both been reported as VUS for RTA syndrome in ClinVar. RTA-related symptoms were not mentioned in the available clinical information for any of our cases with *SLC4A4* variants (Supplementary Table S1). This could reflect the limited clinical information available, a lack of pathogenicity, or that RTA is usually autosomal recessive and we only found heterozygous variants. In the latter scenario, a reduction of sodium bicarbonate cotransporter NBCe1 function could be a contributing factor for HM in some patients due to the pH dependence of many synaptic elements [43]. Notably, two of the patients (cases 120 and 150) had suffered a cerebral bleed/oedema after a relatively minor fall or head injury, which has been observed in some individuals with *CACNA1A* and *ATP1A2* mutations [4,44].

Other members of the family of Na^+^/K^+^ ATPases to which *ATP1A2* belongs have been implicated in disorders with overlapping symptoms. While some *ATP1A2* mutations can cause alternating hemiplegia of childhood (AHC) [45], the majority of cases are caused by mutations in *ATP1A3* [26]. *ATP1A3* mutations can also cause allelic disorders such as rapid-onset dystonia-parkinsonism (DYT12, OMIM #128235) and cerebellar ataxia, areflexia, pes cavus, optic atrophy, and sensorineural hearing loss (CAPOS syndrome, OMIM #601338) [46]. We detected two known *ATP1A3* mutations in two cases (165 and 87), both of which presented with symptoms of AHC (Table S1). Both mutations are recurrent—in a survey of 155 unrelated AHC patients 43% had the *ATP1A3* c.2401G>A (p.Asp801Asn) mutation and 16% had a c.2443G>A (p.Glu815Lys) mutation, which was associated with a more severe phenotype that included intellectual and motor disability [46]. These were historical patient samples referred when *ATP1A2* had been the only gene reported with AHC causal mutations. In retrospect, as the genotype/phenotype correlation seems clear for the *ATP1A3* mutations identified and AHC, such samples should likely not be included for HM gene testing, but rather directly screened for *ATP1A3* variants. However, as migraine-related disorders may have overlapping symptoms, this and other studies show that NGS panel or whole exome sequencing will improve diagnostic rates.

The gene that harboured most rare variants, many of which are predicted to be damaging, was *ATP1A4*. An *ATP1A4* variant (c.1798 C>T; p.Pro600Ser, not found in our study) has only been linked to HM in a single family thus far, and no detailed functional assessments have been done, so it remains unclear as to whether variants in *ATP1A4* are causal for the disorder. *ATP1A4* is mainly expressed in the testes, although low levels appear to be present in other tissues including brain, and a larger transcript is expressed in muscle [47]. The high number of variants detected in this gene is of interest, but may also reflect a low level of constraint on the gene, e.g., in gnomAD the low Z score (0.49) suggests that missense mutations are tolerated (cf *ATP1A2* = 4.77, *ATP1A3* = 6.33) [48]. Further assessment of *ATP1A4* variants in control populations and their functional studies will be required to clarify the role of this gene in HM.

**Figure 3:**
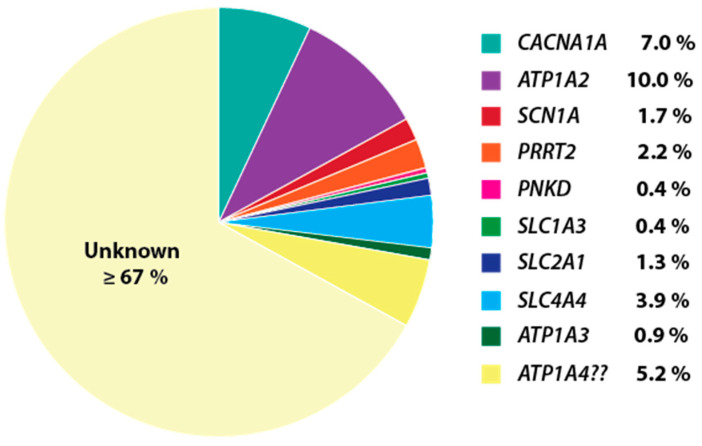
The prevalence of cases referred for HM gene testing with a known or potential pathogenic variant in previously reported genes (% = estimated percentage contribution). *N* = 230 individuals were assessed including cases with *CACNA1A*, *ATP1A2*, and *SCN1A* mutations reported in Maksemous et al. [4], older cases diagnosed by Sanger sequencing of targeted exons, and additional recent samples. A total of 18.7% patients referred for HM testing had known mutations or likely pathogenic variants in the *CACNA1A* (7.0%), *ATP1A2* (10.0%), and *SCN1A* (1.7%) genes. Including mutations and potentially pathogenic variants identified in genes analysed in this study increased the number of cases with a possible genetic diagnosis to 27.8% (not including variants in *ATP1A4*, which remains putative). For the majority of remaining cases, no rare protein-changing variants were detected in HM genes reported to date.

While most HM cases with a molecular diagnosis have mutations in the *CACNA1A*, *ATP1A2*, and (to a lesser extent) *SCN1A* genes, only 18.7% of the patients in our suspected HM cohort appear to have pathogenic coding mutations in these genes (Figure 3) [4], which suggested that mutations in other genes previously linked to HM could account for some of the patients remaining without a genetic diagnosis. Up to an additional ~10% of patients have rare predicted functional variants in the other HM-related genes investigated in this study (Table 1, Figure 3). Thus, *PRRT2*, along with the other genes presented in this study, should be considered when testing for molecular defects in HM cases, as they may harbour mutations in some individuals that present mainly with symptoms of HM. Figure 3 shows the contribution of rare variants detected for each HM gene analysed in our diagnostic cohort. Nevertheless, even in the unlikely scenario that all the rare variants we detected were pathogenic, two-thirds of the cases do not have mutations in any known HM genes.

Our results and others [3,34] suggest that in addition to the HM genes identified to date, the disorder is genetically heterogeneous and additional genes are likely to be involved. Preliminary analysis of our WES data has found potential variants in numerous specific genes that may contribute to the genetic spectrum of HM, including other ion channel gene family members and genes involved in synaptic functions, with further work required to provide supporting evidence. Interrogating WES data in 47 HM patients, Pelzer et al. failed to identify other major HM genes, but noted that most of these cases had a milder phenotype to those with *CACNA1A*, *ATP1A2*, and *SCN1A* mutations [34]. By analysing WES data from various migraine cohorts, Rasmussen et al. found that FHM patients had a high burden of rare frameshift indels in genes involved in synaptic function compared to either familial or sporadic migraine cases [49]. HM has been mostly viewed as an autosomal dominant disorder caused by mutations in ion channel genes, but it may also be caused by mutations in other genes, or by combinations of less penetrant variants, copy number variations, or mutations that affect regulatory regions. Assigning causality to some of these factors will be challenging and better targeted to samples in which other known genes or factors have been ruled out. HM appears to be genetically complex, similar to epilepsy, a disorder in which many genes have been implicated [50], some of which are themselves associated with HM.

Major limitations of this study include a lack of detailed clinical information and unavailability of family members for segregation analysis in many cases. Nevertheless, reporting of identified variants is important, particularly for those that may not be causal on their own, but interact with or modify the effect of other gene variants. Further functional studies, e.g., measuring impact on channel function of particular variants, may add additional evidence of clinical relevance and better understanding of mechanisms. The fact that there is a wide spectrum of variants with few recurrent ones make such studies challenging, although those reported multiple times or in the same gene could be prioritised. Study of multiple cohorts via collaboration will be required to build a comprehensive understanding of the spectrum of variants and genes that contribute to the disorder and allow more nuanced genetic testing in the future. Further analysis of WES data is likely to identify some of these, while whole genome sequencing and more complex analysis may be required to find regulatory variants or copy number variations that may be involved.

Targeted NGS panels or WES makes it much more feasible to scan all genes known to be involved in a particular disorder for causal mutations. Although many neurological disorders can show overlapping symptoms, having a molecular diagnosis can impact on management or treatments. GLUT1 deficiency caused by *SLC2A1* mutations can be treated using a ketogenic diet, and case 189 can be improved on a modified Atkins diet [40]. Carbamazepine is the most frequently used drug in treating PKD and PKD/IC patients, with many patients showing a good response. Some benefit with carbamazepine has been observed in HM cases with *PRRT2* mutations [51]. These findings suggest that targeting treatment to the molecular cause, rather than the manifestation of symptoms, may improve treatment efficacies.

## 5. Conclusions

Using exonic sequencing, we screened a cohort of 187 probands from Australia and New Zealand referred for genetic testing for potential disease-causing variants in all genes previously implicated in HM. No patients were found to have the recurrent *PRRT2* p.Arg217Profs*8 mutation. However, we identified rare missense or potential protein-altering variants in *PRRT2*, *PNKD*, *SLC1A3, SLC2A1*, *SLC4A4, ATP1A3*, and *ATP1A4* in specific cases, suggesting that their screening improves molecular diagnosis for the disorder. Nevertheless, this applied to a minority of the suspected HM patients. As all the cases were also negative for pathogenic mutations in *CACNA1A*, *ATP1A2*, and *SCN1A*, our study shows that more than two-thirds of patients referred for HM genetic testing did not have exonic mutations in any of the known causal genes. Other genes that cause or contribute to HM are likely to be identified from detailed analysis of WES data and will aid in more comprehensive diagnostic screening and subsequent treatment choice.

## Figures and Tables

**Table 1:** Rare functional variants identified by Sanger and targeted analysis of whole exome sequencing data for the *PRRT2*, *PNKD*, *SLC1A3, SLC2A1*, *SLC4A4*, and *ATP1A4* genes in 187 patients referred for genetic testing for hemiplegic migraine, but negative for mutations in *CACNA1A*, *ATP1A2*, and *SCN1A.*

Gene	Patient ID#	Locus	Transcript	Coding	Protein Change	dbSNP	MAF gnomAD (No. Alleles)	MAF gnomAD Eur NF (No. Alleles)	ClinVar (or LOVD) Annotation	SIFT ^a^	Polyphen2 ^b^	Mutation Taster ^c^	Conservation ^d^ PhylopP/ PhastCons
*PRRT2*	224 225	chr16:29824442	NM_145239.3	c.67G>A	p.Glu23Lys	rs140383655	0.0011 (306)	0.0017 (219)	LB, VUS	T	B	P	0.01/0.68
	185 201 211 227	chr16:29825022	NM_145239.3	c.647C>T	p.Pro216Leu	rs76335820	0.007 (1737)	0.010 (1183)	B, LB	D	D	D	3.75/0.99
	433	chr16:29825025	NM_145239.3	c.650G>A	p.Arg217Gln	rs75497546	0.000008 (2)	0.0000092 (1)		D	D	P	1.94/0.75
	137	chr16:29825762	NM_145239.3	c.988G>A	p.Ala330Thr	rs757132796	0.0000042 (1)	0.0000092 (1)		D	PD	D	2.77/1
	261	chr16:29825888	NM_001256442	c.1114C>T	p.Leu372Phe	rs565298585	0.000046 (13)	0.0001 (13)		D	B	P	0.07/0.99
*PNKD*	32	chr2:219209684	NM_015488.5	c.1140_1145delTATGCA	p.Met381_His382del	rs576363906	0.00061 (170)	0.0010 (135)	LB	N/A	N/A	P	N/A
*SLC1A3*	135 155 159 196	chr5:36677083	NM_004172.5	c.657G>C	p.Glu219Asp	rs2032892	0.023 (6697)	0.0069 (887)	B, LB	T	B	P	
	107	chr5:36680556	NM_004172.5	c.1154G>A	p.Arg385His	rs115702388	0.00026 (74)	0.000061 (8)	B, LB	D	D	D	6.24/0.998
*SLC2A1*	109	chr1:43396789	NM_006516.2	c.203C>T	p.Ser68Leu		0	0	VUS	D	B	D	3.79/0.997
	189	chr1:43394924	NM_006516.2	c.929C>T	p.Thr310Ile	rs80359824	0	0		D	D	D	3.47/1
	179	chr1:43394873	NM_006516.2	c.972+7del (intronic)	p.Leu232PhefsTer?	rs531385270	0.00018 (52)	0.00038 (50)	B, LB, VUS	N/A	N/A	D	1.22/0.62
*SLC4A4*	136	chr4:72205146	NM_001098484.3	c.313C>G	p.Pro105Ala	rs768913941	0.000056 (16)	0.000092 (12)		D	B/PD	D	6.09/1
	142	chr4:72306423	NM_001098484.3	c.898A>G	p.Ile300Val	rs747159754	0.000053 (15)	0.00010 (11)		T	B	D	1.72/1
	237	chr4:72316925	NM_001098484.3	c.1229G>A	p.Gly410Glu		0	0		T	B	D	5.76/1
	120 179	chr4:72338589	NM_001098484.3	c.1805A>G	p.Lys602Arg	rs72650362	0.0022 (609)	0.0035 (455)		D	D	D	5.00/1
	202 232	chr4:72363294	NM_001098484.3	c.2051A>T	p.Asn684Ile	rs35891845	0.0016 (443)	0.0029 (368)		T	B	D	2.58/1
	128	chr4:72399974	NM_001098484.3	c.2311C>T	p.Pro771Ser	rs140882617	0.0014 (414)	0.0017 (231)	VUS	T	B	D	2.55/1
	150	chr4:72412109	NM_001098484.3	c.2485C>A	p.Leu829Ile	rs201643562	0.00036 (103)	0.00056 (72)	VUS	D	D	D	4.27/1
*ATP1A3*	165	chr19:42474557	NM_152296.5	c.2401G>A	p.Asp801Asn	rs80356537	0	0	Pathogenic for AHC2	D	D	D	4.83/1
	87	chr19:42474436	NM_152296.5	c.2443G>A	p.Glu815Lys	rs387907281	0	0	Pathogenic for AHC2	D	D	D	4.77/1
*ATP1A4*	228	chr1:160123000	NM_144699.4	c.193G>A	p.Val65Met	rs7549352	0.00087 (246)	0.0015 (194)		D	PD	D	2.14/0.95
	150	chr1:160125005	NM_144699.4	c.378G>T	p.Gln126His	rs370755520	0.0013 (364)	0		D	B	D	2.46/0.93
	161 186	chr1:160125859	NM_144699.4	c.436G>A	p.Val146Ile	rs41288133	0.0022 (623)	0.0041 (539)		T	B	P	0.322/0
	186	chr1:160129260	NM_144699.4	c.722A>G	p.His241Arg	rs151137285	0.0005 (140)	0.0010 (129)		D	B	D	4.453/1
	189	chr1:160133954	NM_144699.4	c.787C>T	p.Arg263Trp	rs146761116	0.00037 (105)	0.00063 (81)		D	D	P	1.41/0.95
	133 144 204	chr1:160133955	NM_144699.4	c.788G>A	p.Arg263Gln	rs76528638	0.014 (3969)	0.0012 (159)		T	B	P	0.83/0.92
	141 188	chr1:160134012	NM_144699.4	c.845C>T	p.Thr282Met	rs144463520	0.001 (307)	0.0020 (268)		D	D	D	5.19/1
	92	chr1:160136403	NM_144699.4	c.1133C>T	p.Thr378Met	rs150693480	0.000045 (13)	0.000061 (8)		D	D	D	5.09/1
	90	chr1:160136459	NM_144699.4	c.1189G>A	p.Ala397Thr	rs147875149	0.000050 (14)	0.0000077 (1)		D	D	D	1.84/1
	144	chr1:160141171	NM_144699.4	c.1622T>G	p.Met541Arg	rs16831482	0.000004 (1)	0.0000087 (1)		D	PD	D	1.63/0.87
	144	chr1:160141525	NM_144699.4	c.1832A>G	p.Lys611Arg	rs79938119	0.0041 (1180)	0.000061 (4)	B, VUS (LOVD)	D	D	D	2.64/1
	96	chr1:160143962	NM_144699.4	c.2053G>C	p.Asp685His	rs144428770	0.00081 (228)	0.0016 (207)		D	D	D	3.49/0.96
	124	chr1:160146341	NM_144699.4	c.2539A>T	p.Thr847Ser	rs145873902	0.00016 (45)	0.00026 (34)		T	B	P	1.38/0.86

ID#, identification number; HM, hemiplegic migraine; dbSNP, Single Nucleotide Polymorphism Database; MAF gnomAD, minor allele frequency in The Genome Aggregation Database (gnomAD); Eur NF, European Non-Finnish; LOVD, Leiden Open Variation Database v3.0; AHC2, alternating hemiplegia of childhood 2. ^a^ Sorting Intolerant from Tolerant (SIFT) variant pathogenicity prediction tool: D, damaging; T, tolerated. ^b^ Polymorphism Phenotyping v2 (Polyphen2) variant pathogenicity prediction tool: D, probably damaging; PD, possibly damaging; B, benign. ^c^ Mutation Taster variant pathogenicity prediction tool: D, disease-causing; P, polymorphism. ^d^ Nucleotide conservation scores derived from: PhyloP, values between 0 and 1, 1 high conservation; PhastCons, values between −14 and +6, >2 high, >4.88 very high conservation.

## Supplementary Materials

The following are available online at https://www.mdpi.com/2073-4409/9/11/2368/s1, Table S1: Characteristics and clinical information for patients carrying potentially pathogenic variants in hemiplegic and familial migraine genes.
