# Supplementary material for: Comprehensive Exonic Sequencing of Hemiplegic Migraine-Related Genes in a Cohort of Suspected Probands Identifies Known and Potential Pathogenic Variants
Source: Cells. 2020 Oct 28;9(11):2368. doi: 10.3390/cells9112368 (PMC7693486; doi:10.3390/cells9112368)
Supplement: Supplementary file 1 [file cells-09-02368-s001.pdf]

Supplementary Table 1. Characteristics and clinical information for patients carrying potentially pathogenic variants in hemiplegic and familial migraine genes

Patient ID#	Sex	Age (years)	Known family history	Gene/s with identified variants	Clinical information, additional comments
32	F	58		*PNKD*	Familial hemiplegic migraine
87	M	19		*ATP1A3*	Alternating hemiplegia of childhood
90	M	18	Y	*ATP1A4*	Benign occipital epilepsy and migraine family - *CACNA1A* and FHM gene testing
92	F	44		*ATP1A4*	No clinical information – FHM gene testing
96	F	40		*ATP1A4*	Hemiplegic migraine
107	M	30		*SLC1A3*	No clinical information – FHM gene testing
109	M	15		*SLC2A1*	Severe hemiplegic migraine, long history vertigo and ataxia as infant.
120	M	1	N	*SLC4A4*	Severe bleed following suspected minor fall. A relatively minor fall which was followed by malignant cerebral oedema, and a relatively small subdural bleed with uncal herniation within half an hour of the fall.
124	F	45	Y	*ATP1A4*	Family with FHM. Patient has severe long lasting symptoms.
128	F	41		*SLC4A4*	Familial hemiplegic migraine, no other information
133	F	70		*ATP1A4*	Familial hemiplegic migraine
135	M	54		*SLC1A3*	Familial hemiplegic migraine
136	F	52	Y	*SLC4A4*	Recurrent migraine attack with hemiplegia, strong history of hemiplegic migraine and a family history of arterial occlusive disease and venous thomboses. Her brother died aged 61 with a coronary thrombosis with a diagnosis of homocystinuria. Pts has a normal homocysteine level. He had occlusive disease of the coronary and the femoral arteries, transient ischemic attacks and had a stroke. The patient's mother was thought to have had a stroke when she was 10 and episodes of ischaemic heart disease when she was a child and again at 38 years. She died with a CVA at 65. Our patient's CT scan was normal and she has had normal skin biopsy for NOTCH3. Therefore could your laboratory test for mutations that cause FHM?
137	F	45		*PRRT2*	Familial hemiplegic migraine
141	F	13		*ATP1A4*	Familial hemiplegic migraine
142	F	29		*SLC4A4*	Episodic hemiplegia with sometimes seizure and encephalopathy
144	M	3		3x *ATP1A4*	Seizure like episodes, Familial hemiplegic migraine
150	F	2	N	*SLC4A4*, *ATP1A4*	Ischaemic stroke after a mild head injury. There is no family history of migraine including hemiplegic migraine.
155	F	16		*SLC1A3*	Frontal epilepsy and psychosis
159	F	3	Y	*SLC1A3*	Patient has had episodes of hemiplegia, encephalopathy, language disturbance as well as drop attacks, positive family history of migraine. FHM
161	F	9	Y	*ATP1A4*	Migraine coma, episode of right sided hemiplegic migraine, past history epilepsy since 3yrs, mother has history of migraine, on episode of transient hemiplegia, maternal uncle said to have "headaches".
165	M	8	Y	*ATP1A3*	Alternating hemiplegia of childhood with family history of migraines including a hemiplegic migraine in his mother.
179	F	50		*SLC2A1*, *SLC4A4*	No clinical information – FHM gene testing
185	M	36	N	*PRRT2*	Hemiplegic migraine with no family history
186	F	50		2x *ATP1A4*	Hemiplegic migraine
188	F	80		*ATP1A4*	Familial hemiplegic migraine
189	M	9	Y	*SLC2A1*, *ATP1A4*	Patient has a severe headache once a week become worse, after a severe episode of headache he wakes up being unresponsive with his speech and paralysis of the right side of the body. This lasts 30 min and then recovers on its own. Strong family history of

					hemiplegic migraine.
196	M	36		*SLC1A3*	Migraine with right sided hemiplegia
201	F	54		*PRRT2*	Hemiplegic migraine
202	M	18		*SLC4A4*	Recurrent migraine headache
204	M	13	N		Migraine-like seizures (initial dizziness, nausea and headache, followed by variably lateralised numbness, stiffness, jerking and weakness, somethimes with aphasia. Headache and persistent hemideficit would continue for a few days. No history of visual symptoms or hemianopia. MRI imaging has shown cortical swelling. He has been on Keppra (increased to 1000mg bd). No family history of migraine, seizures or neurological attacks with hemi-symptoms. Occipital seizure.
211	M	5	Y	*ATP1A4*	Background history of autism, fell on back of head, prolonged recovery with persistent hypotonia and ataxia and ocular motor apraxia. All scans including MRI brain normal, Family history of migraines (hemiplegia and ataxia)
224	F	4		*PRRT2*	Possible CACNA gene with benign toticollis - hemiplegic migraine?
225	M	2	N	*PRRT2*	Channelopathy? Complicated migraine. Episodes of ataxia, vomiting and encephalopathy lasting for 3-4 days. Some of the episodes have been precipitated by intercurrent illness/ No metabolic derangement found. Previously had episodes of intermittent abdominal pain, which continue but are less frequent on a combination of Oxcarbazepine and Gabapentin. SCN9A positive as is his father - however the father doesn't have symptoms. Past history - Paroxysmal episodes of unexplained abdominal pain, sometimes induces by opening of bowels. The child is flushed, stiff and tachycardic during the episode. Cries insolably and hasn't had any response to the usual painkillers. The episode may go on for minutes to half an hour. All gastroenterology, surgical and allergy work up negative. No family history of similar problems.
227	F	39		*PRRT2*	New seizures, right facial droop, persistent headache
228	F	11		*ATP1A4*	Hemiplegic Migraine, Familial Migraine?
232	M	10		*SLC4A4*	No clinical information – FHM gene testing
237	F	54		*SLC4A4*	No clinical information – FHM gene testing
261	F	46		*PRRT2*	Familial hemiplegic migraine
433	F	59		*PRRT2*	Familial hemiplegic migraine
